# Ceramide Metabolism Balance, a Multifaceted Factor in Critical Steps of Breast Cancer Development

**DOI:** 10.3390/ijms19092527

**Published:** 2018-08-26

**Authors:** Victor García-González, José Fernando Díaz-Villanueva, Octavio Galindo-Hernández, Israel Martínez-Navarro, Gustavo Hurtado-Ureta, Abril Alicia Pérez-Arias

**Affiliations:** Departamento de Bioquímica, Facultad de Medicina Mexicali, Universidad Autónoma de Baja California, Mexicali 21000, Baja California, Mexico; jdiaz92@uabc.edu.mx (J.F.D.-V.); octavio.galindo@uabc.edu.mx (O.G.-H.); israel.martinez.navarro@uabc.edu.mx (I.M.-N.); gustavo.hurtado@uabc.edu.mx (G.H.-U.); abril.alicia.perez.arias@uabc.edu.mx (A.A.P.-A.)

**Keywords:** ceramides, meta-inflammation, breast cancer

## Abstract

Ceramides are key lipids in energetic-metabolic pathways and signaling cascades, modulating critical physiological functions in cells. While synthesis of ceramides is performed in endoplasmic reticulum (ER), which is altered under overnutrition conditions, proteins associated with ceramide metabolism are located on membrane arrangement of mitochondria and ER (MAMs). However, ceramide accumulation in meta-inflammation, condition that associates obesity with a chronic low-grade inflammatory state, favors the deregulation of pathways such as insulin signaling, and induces structural rearrangements on mitochondrial membrane, modifying its permeability and altering the flux of ions and other molecules. Considering the wide biological processes in which sphingolipids are implicated, they have been associated with diseases that present abnormalities in their energetic metabolism, such as breast cancer. In this sense, sphingolipids could modulate various cell features, such as growth, proliferation, survival, senescence, and apoptosis in cancer progression; moreover, ceramide metabolism is associated to chemotherapy resistance, and regulation of metastasis. Cell–cell communication mediated by exosomes and lipoproteins has become relevant in the transport of several sphingolipids. Therefore, in this work we performed a comprehensive analysis of the state of the art about the multifaceted roles of ceramides, specifically the deregulation of ceramide metabolism pathways, being a key factor that could modulate neoplastic processes development. Under specific conditions, sphingolipids perform important functions in several cellular processes, and depending on the preponderant species and cellular and/or tissue status can inhibit or promote the development of metabolic and potentially breast cancer disease.

## 1. Introduction

A chronic excess of triglycerides in the body, specifically within adipose tissue, can lead to its saturation and promote the release of free fatty acids (FFA) to blood circulation. These lipids could be transported by serum albumin and lipoproteins, promoting their accumulation in tissues such as liver, pancreas, and skeletal muscle, which triggers alterations in several signaling pathways associated with energetic metabolism and cell cycle regulation [1]. Importantly, saturated fatty acids (SFA) have been described as modulators of gene expression targets involved in the metabolism of sphingolipids [2]. For instance, the increase of cytoplasmic palmitoyl-CoA by lipogenesis and cell internalization could drive fatty acids toward ceramides synthesis in endoplasmic reticulum (ER) through the de novo pathway. Therefore, ceramides modify several metabolic pathways generating a cellular imbalance, for example C16 ceramide could block the activity of the electron transport chain (ECT) by inhibition of complex IV, inducing the generation of reactive oxygen species (ROS) [3]. In addition, an increase in mitochondrial ceramides contributes to the sensitization of the IP3R, favoring the release of Ca^2+^ from ER to the mitochondria [4].

**Figure 1:**
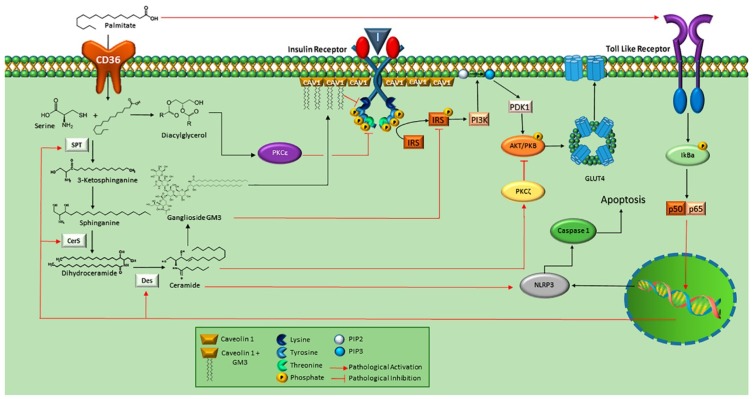
Metabolic implications associated to ceramide synthesis. Free fatty acid (FFA) such as palmitic acid is internalized through CD36, and ceramide synthesis is stimulated by serin-palmitoyl acyltransferase (SPT) through the addition of serine to produce sphinganine. Then, is converted into dihydroceramide by CerS and finally to ceramide by Des. Ceramides promote inhibition of Akt/PKB signaling by PKC function. Ceramides can be turned to GM3 which inhibits INRS, blocking insulin signaling. GM3 accumulation promotes the dissociation of IR/Cav-1 complex. Likewise, palmitic acid can function as a ligand for TLR-4, activating its signaling cascade that leads to the expression of genes encoding enzymes such as SPT, CerS, and Des. On the other hand, palmitic acid can be metabolized to DAG, which stimulates PKCε, activated PKCε phosphorylates Thr 1160 of INSR, causing its inhibition. Finally, ceramides could activate NLRP3 and lead to apoptosis by Caspase-1-dependent pathway. Image adapted from references: [9,10,11,12,13].

Accumulation of triglycerides and intermediate molecules such as ceramides and diacylglycerols (DAGs) are a consequence of chronic overnutrition [5,6]. Therefore, DAGs, ceramides and SFAs induce chronic inflammatory conditions, originating cellular deleterious effects [7]. An increase in the synthesis of C16-ceramides is associated with obesity and development of diabetes mellitus type 2, inhibiting fatty acid oxidation [8] (Figure 1). Cytosolic DAGs are associated with activation of hepatic PKCε [9], which has been demonstrated to phosphorylate the insulin receptor (INSR) in Threonine-1160 leading to inhibition of IRS kinase activity [10]. Thus, activation of hepatic PKCε induced by DAGs in the pathogenesis of non-alcoholic fatty liver disease has been related to hepatic insulin resistance [9]. In addition, activation of TLR-4 by palmytic acid could trigger a cascade of intracellular signaling mediated by phosphorylation of IkBa, which induces the activation of p50 and p65, and the expression of serin-palmitoyl acyltransferase (SPT), ceramide synthase (CerS) and dihydroceramide desaturase, key enzymes in the synthesis of ceramides [11]. Likewise, the increase in intracellular ceramides could trigger the activation of NOD-like receptor family pyrin domain containing 3 (NLRP3) [12], complex that integrates the inflammasome. Indeed, inflammasome functions as a putative sensor of ceramides; therefore caspase-1 is activated by this mechanism [13]. Evidence shows a close relationship among the role of ceramides with the development of metabolic pathologies (Figure 1).

In addition, an increase in the synthesis of ceramides associated with ER stress promotes the flow of lipids among ER and mitochondria through the structures such as mitochondria-associated ER membranes (MAMs) and multivesicular endosomes (MVEs), moreover these structures are involved in mechanisms such as ceramide synthesis, ATP flux between ER and mitochondria, as well as exosome-secretion [14]. In an important way, exosomes are vesicles that perform key functions in processes of autocrine, paracrine and endocrine communication. Specifically, MVEs are composed of intraluminal vesicles and participate in internalization of macromolecules and vacuolar/lysosomal hydrolase delivery [15]. Once molecules are incorporated in these vesicles, they have two pathways, degradation or secretion as exosomes by fusion with the plasma membrane, a process that is regulated by the endosomal sorting complex required for transport (ESCRT-I). Indeed, exosomes have been described to be enriched in ceramides; consequently, exogenous treatment with a sphingomyelinase inhibitor reduced the releasing of exosomes, as well as in the depletion of neutral sphingomyelinase-2, which suggests that ceramide metabolism is implicated in biogenesis of exosomes from intraluminal vesicles [14]. Furthermore, has been described that MVEs formation, and consequently, exosome secretion can be related with breast cancer, phenomenon proposed as a cellular stress response [16].

Ceramides play critical roles in the alteration of membrane structures, for instance, these lipids are a factor that triggers the formation of channel-like structures in the outer mitochondrial membrane (OMM), modifying conditions such as permeability and ionic concentration; thus, these conditions could facilitate the flux of pro-apoptotic proteins such as cytochrome-C, Smac/Diablo, and Omi/HtrA2, molecules of apoptosis intrinsic pathway. Paradoxically, ceramides function in apoptosis development has allowed a more complex visualization of them, such as their role in cell differentiation and proliferation signaling pathways [17,18]. Ceramides have been described as regulatory lipids that modulate various sides of cell growth, survival, senescence, and apoptosis in cancer [19,20]. Wherein, a correlation between the levels of ceramides and the tumor stage has been demonstrated, ceramides metabolism has also been associated to chemotherapy resistance [19,20]. Then, ceramides play important roles in the regulation of cancer progression, and are linked to their function in meta-inflammation, a condition that associates overnutrition and obesity with a chronic low-grade inflammatory state [21,22], representing a key factor in the regulation of pathological processes. Therefore, in this work, we performed an analysis of the state of the art about the multifaceted role of ceramides in several pathological processes, with a focus on lipid metabolism and breast cancer. 

## 2. Pathways of Ceramide Synthesis

Ceramide synthesis can be carried out by means of hydrolysis of sphingomyelin, de novo synthesis, and the salvage and recycling pathway; therefore, there is a continuous flux of these lipids in cells. For instance, de novo synthesis is activated by increased saturated fatty acid accumulation, wherein palmitoyl-CoA and serine are conjugated by SPT, producing the unstable molecule 3-ketosphinganine, which is rapidly converted to dihydro-sphingosine that undergoes N-acylations by the CerS, producing dihydroceramides. The function of CerS is considered a critical regulatory step, localized in ER and nuclear envelope. Later, the dihydroceramides are transformed by the dihydroceramide desaturase (DES 1 and 2) into ceramides. Subsequently, ceramides are delivered to the Golgi apparatus by vesicle transport, or through carrier proteins such as ceramide transfer protein (CERT) [23,24], which is the only described protein capable of transferring vesicle-independent ceramides among organelles [25].

CerS are composed of six variants whose function lies in the condensation of acyl-CoA of different lengths to its sphinganine. Differences among CerS variants are associated with transmembrane topology, specificity for long chain fatty acids, and tissue distribution [26], each CerS changes during development and is expressed according to the cell type [26]. CerS1 and CerS4 generate C18–C20 ceramides, CerS5 and CerS6 generate C14–C16 ceramides, CerS2 selectively generates C22–C24 ceramides [27,28], and CerS3 mediates the synthesis of very-long chain C28–C32 ceramides with polyunsaturated fatty acids [29]. The activity of each CerS is determined by different post-translational modifications, as well as homo and heterodimerization.

Sphingomyelin is the main sphingolipid constituent of the plasmatic membrane, and ceramides generated by this pathway are produced by the hydrolysis of the phosphocholine head by the action of sphingomyelinase (SMase), which can be activated by extracellular signals such as oxidative stress, inflammation, and ionizing radiation [23]. Likewise, the salvage pathway can be activated by oxidative stress, this process begins in lysosomes or late endosomes wherein sphingomyelin and glycosphingolipids are hydrolyzed by the acid sphingomyelinase and β-glucosidase-1, respectively. Sphingolipids are released from these structures in the form of sphingosine and fatty acids, reaction catalyzed by acid ceramidase, this pathway could be regulated by PKCδ which activates sphingomyelinase. Furthermore, the recycling pathway involves deacylation of exogenous short chain ceramides by ceramidase to produce sphingosine, these pathways converge to obtain a product in common: ceramide produced by ceramide synthase [30]; likewise the recycling pathway has been involved in apoptosis [31]. Evenmore, gangliosides can function as precursors of ceramides, through the function of plasma membrane sialidase, to become sialosyl lactosyl ceramides [23].

Importantly, ceramide synthesis is activated by different cellular stimuli, including increased intracellular concentration of postprandial palmitate, hypoxia, intracellular stress, activation of the neutral sphingomyelinase [24,25], as well as inflammatory stimuli mediated by TLR-4 in response to TNFα and INFγ signaling, inducing the expression of enzymes such as SPT [32]. Therefore, the activation of ceramide synthesis due to meta-inflammation as a result of overnutrition, could generate a deleterious circle by the activation of proinflammatory and pro-apoptotic signaling in metabolic tissues, modifying organelles structure, then the pathological state is amplified [33].

## 3. Ceramides, Association with Endoplasmic Reticulum and Mitochondria

**Figure 2:**
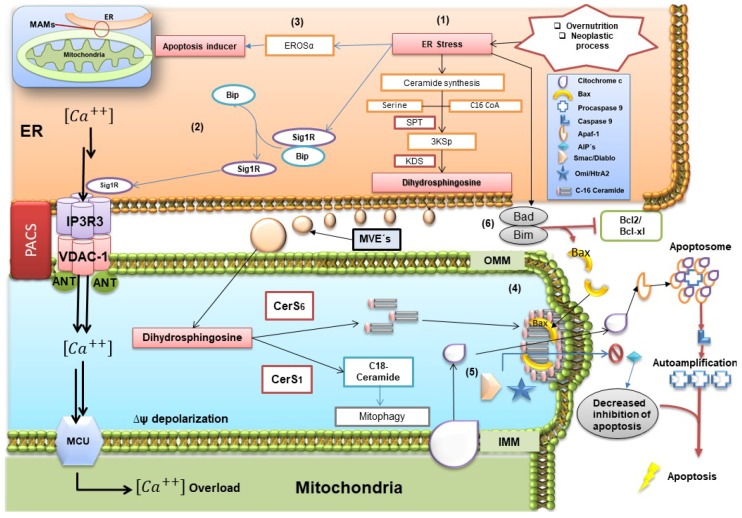
Overview of endoplasmic reticulum (ER) and mitochondria interactions. ER stress phenomenon promotes the activation of the de novo ceramide pathway, producing an increase in the exportation of dyhidrosphingosine and enzymes such as CerS1 and CerS6 through mitochondria-associated ER membranes (MAMs) or multivesicular endosomes (MEVs) to mitochondria (1), this originates the cleavage of Sig1R/Bip complex, Sig1R binds to IP3R3/VDCA-1 allowing the flow of Ca^2+^ through the ER to the inter-membranal space of the mitochondria by MCU (2), it can cross the inner mitochondrial membrane, producing a supersaturation in the mitochondrial matrix. This condition facilitates the activation of apoptosis by means of CHOP-dependent EROSα (3). In inter-membrane space, C16 ceramide leads to synthesis and assembly of pores that synergistically with Bax are translocated to OMM (4), this modifies the permeability of the membrane and allows the output of cytochrome C and proteins as SMAC/DIABLO and Omi/Htra (5). Later, cytochrome-C is associated with Apaf-1, promoting the change from procaspase-9 to caspase-9, leading to apoptosome formation. The complex Bad/Bim inhibits the antiapoptotic action of Bcl-2 and Bcl-xL, which in turn are associated with preventing the oligomerization of Bax with the pores, as well as inhibition of cytochrome C release (6). Image adapted from references: [4,14,18,37,38,39].

Ceramide hydrophobicity represents an energetic barrier that prevents free movement among membrane structures; for instance, communication of ER and mitochondria requires physical contact of membrane proteins that facilitate the flow of molecules. Within structure of MAMs, enzymes involved in lipid biosynthetic pathways of ceramides and glycosphingolipids have been described, for instance, ceramide synthase, ceramide glucosyltransferase, glucosylceramide galactosyltransferase, and sialyltransferase [34]. Physiologically, there is a 5–20 % area of contact between ER and mitochondria, which suggests a considerable flow of molecules between these organelles [35,36] (Figure 2).

ER-mitochondria associations are not only restricted to synthesis and lipid flow: even more mitochondrial ATP transfer to the ER is critical for chaperone function in the activity of unfolded protein response (UPR). One of chaperones, BiP (Grp78), recruits the adaptor protein TRAF2 in cytoplasmic domains and activate ASK-1 and JNK kinase, which could target mitochondria through various signaling pathways such as phosphorylation of Bim, and Bad [40,41], proteins of the Bcl-2 family involved in cell progression pathways and apoptosis [42]. In an important way, by decreasing the fluidity of the mitochondrial membrane, ceramides attenuate the stability of MAMs, which are structures needed for tumor cells adaptation under conditions of metabolic stress [43] (Figure 2).

In this sense, phosphofurin acidic cluster sorting protein 2 (PACS-2) is implicated in ER-mitochondria tethering, which regulates MAMs formation [37]. The function of PACS-2 is dependent of Akt phosphorylation (Figure 2), which enables PACS-2 to be retained in MAMs [44]. Likewise, PACS-2 interacts with calnexin, a regulator of ER-mitochondria Ca^2+^ flux [45,46], and blocks BAP31 processing mediated by caspase 8 [37]. This interaction is the basis of PACS-2-mediated ER-mitochondria tethering, wherein BAP31 interacts with the mitochondrial Drp1 docking protein Fis1 [47]. However, this complex is associated with procaspase-8 under conditions of cell stress where the mature form of caspase-8 triggers the formation of the BAP31 p20 fragment, an activator of mitochondrial fission [48]. Likewise, in cytoplasm, p53 can be located within the MAMs, wherein a fraction of p53 could stimulate the activity of the Sarco/ER Ca^2+^ ATPase pump, regulating Ca^2+^ levels in ER. In fact, under pro-apoptotic conditions, its downward activity causes an overload of Ca^2+^ inside the mitochondria, which is a factor of formation of mitochondrial permeability transition pores [4].

Intracellular increase of ceramides modifies several signaling pathways that maintain cell homeostasis [49]. For instance, longer acyl chain ceramides decrease membrane fluidity of mitochondria [50]. Specifically, the presence of two species of ceramides *N*-acetyl-d-erythro-sphingosine C2 and *N*-palmitoyl-d-erythrosphingosine C16, has been associated together with other proteins to formation of channel-like structures, then increasing permeability of OMM. These structures could facilitate the movement of proteins from the inter-membrane space to the cytoplasm, such as the inducing factor of apoptosis, cytochrome C, procaspases, and several heat shock proteins [51,52]. Nevertheless, this proposal has been questioned; moreover, Lee et al. (2011) [53] propose that ceramides facilitate the effect of Bax, a pro-apoptotic protein, which directly could trigger the pore formation on OMM [53]. 

Experimentation on mitochondria of hepatocytes deleted of Bax showed increasing levels of cytochrome C releasing under Bax treatment than stimulation with C16 ceramide alone, even at high doses. In addition, minimal doses of C16 ceramide and recombinant Bax, potentiated the release of cytochrome C. Therefore, C16 ceramide is not considered a direct inducer of increased permeabilization of OMM, while it improves the insertion of Bax on OMM [53]. Although the release of these proteins promotes the activation of several caspases and DNases, other mitochondrial effects appear, such as the increase in ROS, alterations in calcium homeostasis of MAMs, modifications of several components of the ECT, as well as reduction in ATP concentration with the consequent collapse of inner mitochondrial membrane (IMM) potential. 

While ceramides are lipids capable of inducing the destabilization of OMM, leading to increased membrane sorting and inducing gel/fluid phase separation possibly to form channels, the characterization of these channel-structures shows that only proteins could have the stability to form these structures. Considering that pore formation, as the main mechanism of mitochondrial permeabilization, has been questioned due to a thermodynamic barrier. Even more, the concept of an increased permeability induced by a surface mismatch between the two mitochondrial monolayers has been proposed [54]. Notwithstanding, in vitro studies with liposomes treated with C16 ceramides and supported by electron microscopy and molecular dynamic simulations, have suggested the pore structure formation [55]. In this proposal, ceramide molecules are organized in columns arranged in anti-parallel way originating a cylindrical shape spanning the hydrophobic interior of the OMM [52,56,57]. These phenomena could be dependent on the long chain and the degree of unsaturation of acyl chain of ceramides [4,38]. Nowadays, the debate on the proposal that explains this process in function of thermodynamic feature, still remains.

Likewise, in mitophagy, a physiological process in which cells eliminate dysfunctional mitochondria, ceramides have been described that induce mitophagy, targeting mitochondria that contains LC3B-II autophagolysosomes [58]. Specifically, C18 ceramides have emerged as tumor suppressors. CERS1 expression generating C18 ceramides mediates the localization of ceramide on the OMM, leading to mitophagy. Target mitochondria through LC3—an important component in the formation of autophagosome which is conjugated with phosphatidyl-ethanolamine through the C-terminal domain forming LC3B-II [58,59]—binds ceramides on the mitochondrial membrane upon DNM1L/DRP1, leading to inhibition of mitochondrial function and oxygen consumption [58]. In this sense, the interaction LC3B-II-ceramide involves the central hydrophobic domain of the protein [60].

## 4. Role of MAMs in Neoplastic Pathology

The role of MAMs has been considered a fundamental feature of the anti-apoptotic effect in neoplastic cells, mainly due to the control of Ca^2+^ flux, and the handling of ROS balance, generated by uncontrolled growth and/or antineoplastic therapy [43]. Moreover, the localization of oncogenic and/or tumor suppressor proteins in these special areas of contact ER/mitochondria, participating in the physiological signaling pathways, have direct implications in the development of neoplastic pathology [43]. Currently, several mechanisms have been associated with the function of MAMs in several types of cancer, such as breast, lung, prostate, hematopoietic, and/or lymphoid neoplasias. For instance, the sigma 1 receptor (S1R) in normal tissue is associated with chaperone protein BiP/GRP78 within the area of MAMs [41]; in contrast under conditions of stress it is uncoupled from the BiP, to bind IP3R3, liberating Ca^2+^ from the ER to the MAMs. On the other hand, S1R is translocated under chronic stress towards the periphery of ER, and thereby attenuates the cell death signal that would generate Ca^2+^ overload in mitochondria and then, ER Ca^2+^ depletion during chronic conditions [61].

MAMs perform microzones of anchoring enzymes that maintain oxidative homeostasis, such as endoplasmic reticulum oxidoreductase-1 alpha (ERO-1α), with which overexpression in tumor tissue is associated with poor prognosis [62]. In addition, a strong correlation among the levels of ERO-1α and programmed cell death-ligand 1 (PD-L1) is proposed, allowing the escape of the immunological attack, and confining an immunosuppressive phenotype [63]. However, ERO-1α also participates in cell death mediated by activation of compound 1 activator of procaspase (PAC-1), inducing apoptosis in several tumors [39]. In this sense, ERO-1α shows a dual role in neoplastic pathology, previously mentioned in the implication of oxidative balance and the presentation of immuno-tolerance with anti-apoptotic characteristics, in contrast to cell death via activation of PAC-1 pro-apoptotic feature [39]. In addition, within the structure of MAMs, the PERK arm of UPR is localized [64], its deficiency showed an association with tumors of low proliferation, because PERK triggers mechanisms that maintain redox balance [65].

In colon and breast cancer, ATAD3 has been described as functioning in conjunction with GRP78 within the MAMs, to keep the stability of WASF3, one function of which is the activation of actin polymerization, participating in the metastatic phenomenon, and leading to a bad prognosis [66]. Interestingly, ATAD3 has been pinpoint in the vicinity of the lipid transfer zone between ER and mitochondria [67], the aforementioned is important for maintain the integrity of the lipid bilayer of the mitochondria [68,69]. In lung cancer, the expression of variants of the Bcl-2 family has been described, for instance the presence of Bcl-2 [70,71] and Bcl-XL [72] is associated with an anti-apoptotic phenotype in non-small cell lung carcinoma. On the other hand, myeloid cell leukemia-1 (Mcl-1), another member of the Bcl-2 family within MAMs, activates the voltage-dependent anion-selective channel 1 (VDAC-1), increasing the flux of Ca^2+^, then cell migration [73].

In addition, hexokinase-2 (H2K) is over-expressed in cancer cells, wherein glycolysis has been described to show an important role in the initiation and neoplastic development in models of mice with K-Ras driven lung cancer and Her2/Neu-driven breast cancer [74]. However, the phosphorylation of H2K dependent of Akt causes the translocation of H2K to the MAMs, wherein maintains interaction with VDAC-1, in this manner H2K takes advantage of the emerging ATP of the channel formed by VDAC-1, catalyzing the first reaction of glycolysis, in this way contributes to the Warburg effect [75], a metabolic adaptation to hypoxic microenvironment due to lack of blood supply, and fundamental in the survival neoplastic during early stages.

In this sense, lactate produced by anaerobic glycolysis in Warburg effect generates an acid microenvironment that activates the VEGF signaling pathway, a proper condition to tumor growing [76]. This hypoxic condition leads to activation of HIFα, which, in collaboration with c-Myc activate pyruvate dehydrogenase kinase 1 [77], reduces mitochondrial oxygen consumption and promotes anaerobic glycolysis, then lactate synthesis [78]. Recently, MAMs have been proposed as regulators of cancer cells metabolism due to their function in Ca^2+^ exchange between ER and mitochondria, which is needed for oxidative phosphorylation [79]. Therefore, alteration of MAMs could lead to modifications of Ca^2+^ exchange and ATP production [80]. Moreover, some proteins within the MAMs have been proposed as potential regulators of tumor cell metabolism, for instance, TMX1 is related with glycolysis and oxidative phosphorylation balance [80], mitofusin-2 as an important regulator on MAMs contact has been associated to pro-apoptotic and anti-proliferative signaling [81], and PACS-2 proposed as a tumor suppressor [82].

While MAMs connect membrane surfaces between mitochondria-ER, these associations harbor multiple enzymes and proteins, which allow cell homeostasis preservation and, thus, survival in neoplastic pathology [43]. MAMs represent a focus of therapeutic target for antineoplastic therapy, due to its associations with responses to oxidative stress and harmful microenvironments, favoring resistance to pro-apoptotic stimuli in cancer cells [83]. Indeed, the use of ceramides as coadjuvant therapy with different antineoplastics, is a novel treatment option due to its multifactorial participation in the structure and function of MAMs [84]. In line with this notion, ceramides are bioactive lipids that modulate processes such as proliferation and cell cycle, key events in the development of cancer [19,20]. Various reports have shown that cancer cells strictly modulate ceramide metabolism [19,20,85]. For instance, sphingolipid metabolism has been reported to regulate cancer development, progression, metastasis and resistance to therapy, some mechanisms have been described to explain how ceramides could regulate these processes specifically in breast cancer [19,20].

## 5. Ceramides and Breast Cancer

Ceramide-1-phosphate and glucosylceramides regulate proliferative processes, while dihydro-sphingosine, ceramides, and sphingosine promote apoptosis [19]. While, the effects of ceramides in various cell types are pleiotropic, functioning as inhibitors of cell proliferation, ceramide levels are down-regulated in most types of cancer via decreasing levels or activities of enzymes involved in ceramide synthesis, and/or increasing the activity of enzymes associated with degradation. For instance, in breast cancer cell lines, ceramides promote cell cycle arrest by mechanisms such as the induction of the dephosphorylation of the Rb retinoblastoma protein, the activation of the cyclin-dependent kinase inhibitor p21, and the inhibition of the cyclin-dependent kinase CDK2 [86,87], additionally to promotion of pore formation mechanisms in OMM [88]. Indeed, taking into account the inducer effect of apoptosis in breast cancer, ceramides are considered a tumor suppressor lipid [86,89,90].

**Figure 3:**
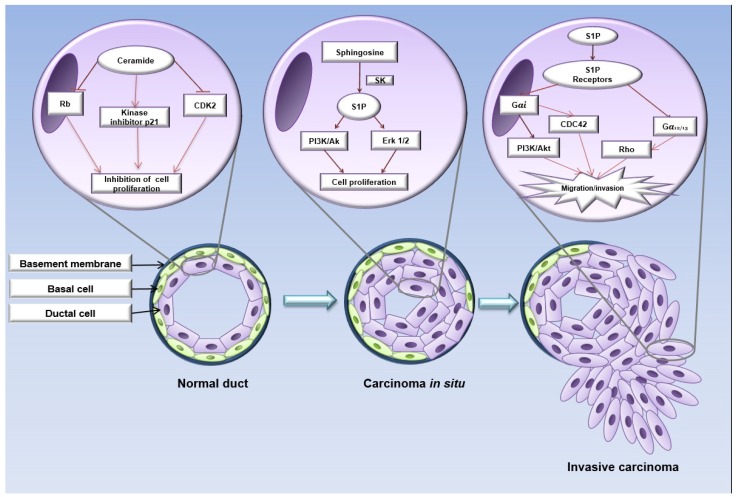
Schematic view of sphingolipid role in breast cancer progression. Structurally, the mammary ducts are composed of basal membrane, a layer of myoepithelial cells and another of luminal ductal cells. In the normal mammary epithelium, ceramides negatively regulate the Rb and CDK2 proteins, inhibiting cell proliferation. In carcinoma in situ, luminal tumor cells present an abnormal metabolism of ceramides, where sphingosine-1-phosphate (S1P) through S1PR promotes activation of the PI3K/Akt and MAPK pathways, inducing cell proliferation. The loss and/or degradation of the basement membrane allow the tumoral cells to migrate and invade the surrounding tissue. In this stage, S1PR promotes GTPases activation such as Rho and CDC42, inducing tumor invasion and consequently, metastasis. Image adapted from references: [12,19,92,102].

In this sense, acid ceramidase is an enzyme that deacylates ceramides in the ceramide metabolic pathway to sphingosine, which is further phosphorylated by sphingosine kinase (SphK) to generate sphingosine-1-phosphate (S1P). Interestingly, ceramides have been shown to induce apoptosis, whereas S1P could regulate cell survival, proliferation, and angiogenesis in cancer cells [19,91]. S1P is described to exert multiple responses, such as proliferation, survival, and cytoskeletal rearrangement, via its G protein-coupled receptor (GPCR) in several cell types, promoting the activation of Rho and CDC42 GTPases, and MAPK, PI3K/Akt pathways. S1P is synthesized from sphingosine by SphK; two isoforms of mammalian SphK (SphK1 and SphK2) have been characterized. Likewise, the S1P has been shown to accumulate in the tumor microenvironment [19,92] (Figure 3).

Therefore, the role of sphingolipids in the development of breast cancer has been controversial, however cancer cells that maintain an active proliferation specifically present low levels of ceramides, by up-regulating enzymes that metabolize these lipids, resulting in conditions of resistance to apoptosis [85,93]. Previous studies described different expression profiles of the sphingolipids enzymes in cancerous tissue relative to normal tissue. Schiffmann et al. showed that endogenous ceramide level was significantly elevated in malignant breast tissue, indicating that elevation of sphingolipid levels correlates with disease status [94]. Particularly, the mammary tumor tissue showed high levels of C16:0-Cer, C24:1-Cer and C24:0-Cer in comparison with non-tumoral tissue, and this increase was associated with higher expression of ceramide synthases CerS2, CerS4 and CerS6. Interestingly, levels of C18:0-Cer and C20:0-Cer were associated with positive tumors for the estrogen receptor, while high levels of C16:0-Cer were linked with metastasis to lymph nodes [94,95].

On the other hand, increasing ceramide levels using ceramide analogs effectively induce apoptosis of breast cancer cells. The stimulation of breast cancer cell lines MCF7 and MDA-MB-231 with the analog of ceramide (2*S*,3*R*)-(4*E*,6*E*)-2-octanoylamidooctadecadiene-1,3-diol promotes apoptosis by translocation of phosphatidylserine to the outer monolayer of the cell membrane and deregulation of mitochondrial membrane potential and released of cytochrome C [96,97]. Therefore, ceramides have been proposed as a therapeutic target for specific types of cancer by synergism with several drugs, as potent tumor suppressors, and acting as second messengers in signal transduction and regulation of proliferation, differentiation, senescence, and apoptosis [85,93]. Chemotherapeutic drugs can induce the endogenous accumulation of ceramides, promoting cancer cells apoptosis, evidence suggests that modulation in the lipid metabolism may be effective to improve tumor cells susceptibility to antineoplastic drugs. Indeed, several chemotherapeutic agents increase the synthesis of ceramide through the activation of SMases or de novo pathway, such as gemcitabine, camptothecin, etoposide, cisplatin, fludarabine, and daunorubicin [98,99]. Furthermore, anti-tumoral treatment modulates the expression of the receptors for S1P. In MCF7 breast cancer cells, tamoxifen and medroxyprogesterone treatment induces an increase of S1P2 expression, while this same treatment induces a down-regulation of S1P3 [100]. However, the biological mechanism that causes this phenomenon has not been described. For instance, treatment of P388 and U937 leukemia cells with daunorubicin promotes apoptosis by increasing ceramide levels by de novo pathway [101]. Subsequently, in the next chapters we are going to analyze the critical steps in cancer progression and the role of ceramide metabolism.

## 6. Involvement of Ceramides and Sphingolipids in the Epithelial-Mesenchymal Transition (EMT)

EMT is an important process involved in embryonic development, wound healing, fibrosis, and breast cancer metastasis [103,104]. The EMT process implies that epithelial cells progressively acquire a mesenchymal phenotype. It is characterized by a decrease in E-cadherin and cytokeratin (CK) 8/18 levels, loss of apical-basal polarity, junctions dissociation, cytoskeleton reorganization, an increase in N-cadherin and vimentin levels [103], as well as matrix degradation and an enhanced ability for cell migration and invasion. Cells that have undergone all these modifications have transitioned from an epithelial to a mesenchymal phenotype [103,104].

For instance, in mouse mammary epithelial cells under the EMT process, a modulation in the metabolism of gangliosphingolipids has been determined. Galpβ1-3GalpNAcβ1-4Galpβ1-4Glcpβ (Gg4) is involved in the regulation of EMT, since the concentration of this lipid is reduced in transdifferentiate cells into mesenchymal cells. In addition, Gg4 forms complexes with proteins such as E-cadherin and β-catenin, preventing its degradation and favoring the epithelial phenotype [99]. Additionally, the differential expression of enzymes related to the ganglioside metabolism observed in EMT is associated with the regulation of transcription factors such as Snail1, ZEB1, and Twist, key EMT-inducing proteins [105].

Likewise, mammary epithelial cells under EMT showed a decrease in the levels of Ceramide C16:0, as well as CerS6. Mammary epithelial cells treated with TGF-β have decreased levels of CerS6, a phenomenon observed in patients with triple negative breast cancer (TNBC) mesenchymal cells [106]. The decrease of C16:0 ceramide levels in patients with TNBC are associated with migratory and invasive capacity of tumor cells. In addition, the down-regulation of CerS6 in tumors is associated with mesenchymal phenotype, as well as aggressive malignancies and poor prognosis [106].

**Figure 4:**
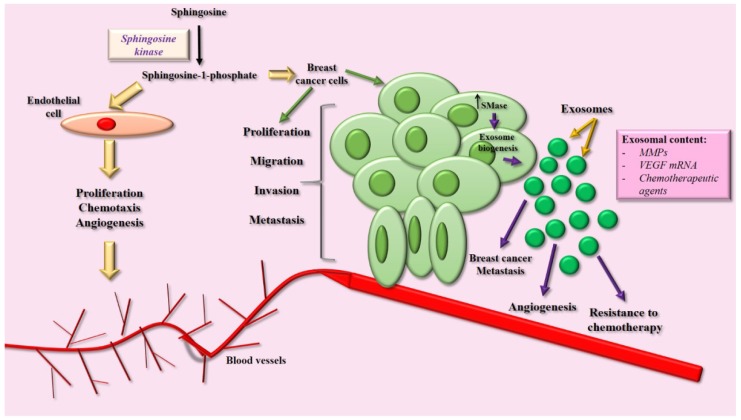
Ceramides and sphingolipids in breast cancer progression. S1P is a critical molecule involved in the modulation of angiogenesis. In endothelial cells, it has been demonstrated that S1P promotes proliferation, migration, and chemotaxis, resulting in the generation of intra-tumoral blood vessels that facilitate the process of metastasis. Moreover, S1P induces migration and invasion of breast cancer cells, promoting the process of tumor intravasation. Finally, in tumor cells, the SMase enzyme shows high activity, promoting the biogenesis and secretion of exosomes, extracellular vesicles that modulate angiogenesis, metastasis, and resistance to chemotherapy. Image adapted from references: [14,120,132,136].

As previously described, S1P is a bioactive lipid that regulates several responses including inflammation, survival, and cell migration [102]. Forming complex with their respective receptors (GPCRs), S1P promotes the activation of non-receptor kinases such as FAK, Src, as well as GTPase Rac, regulatory proteins of cell migration and invasion [102]. In human mammary non-tumorigenic epithelial cells MCF10A, a cellular model for the study of EMT, treatment with S1P induces the activation of the S1P3 receptor, promoting the activation of Erk ½, Rac, Akt, and PI3K, and an increase in the expression and secretion of MMP-9. Moreover, S1P induces an increase in migratory and invasive capacity of mammary epithelial cells, promoting an EMT-like process in MCF10A cells [102] (Figure 4). 

Recently, a study performed in human breast cancer patients showed that ceramide levels are elevated in breast tumor tissue compared to adjacent tissue and/or non-tumor tissue [90]. In addition, the enzymes involved in the de novo pathway, in the salvage and the sphingomyelin pathways are overexpressed in cancerous tissues, accompanied by an increase in the levels of multiple ceramides (C14: 0, C16: 0, C18: 1, C18: 0, C20: 0, C22: 0, C24: 1, C24: 0, C26: 1 and C26: 0), increase in the levels of monohexocylceramide species (C14: 0, C16: 0, C18: 1, C18: 0, C20: 0, C22: 0, C24: 1, C24: 0, C26: 1 and C26: 0), and an increase in sphingomyelin levels (C14: 0, C16: 0, C18: 1, C18: 0, C20: 0, C22: 0, C24: 1, C24: 0, C26: 1 and C26: 0), as previously mentioned these are related with an aggressive phenotype [90].

Interestingly, the metabolism of ceramides is associated with invasiveness and metastasis. For example, key regulatory enzymes in the ceramides and sphingolipid metabolism such as glucosylceramide synthase [26,107], acid ceramidase [108,109], and ceramide kinase [110,111] are overexpressed in mammary tumor tissue and/or breast cancer cell lines, suggesting a strong association between ceramides and their intermediates on tumor progression. Therefore, this broadens the understanding of the role of this class of lipids.

## 7. Ceramides and Sphingolipids and Their Implication in Breast Cancer Metastasis

In mammary tumor progression, the development of new blood vessels, denominated tumor angiogenesis, is a critical process. In this sense, S1P and S1P receptors have been described as key regulators of this process [112]. It has been shown that S1P induces an increase in intracellular calcium concentrations and migration of HUVECs cells through the activation of the S1P receptor [113]. Additionally, S1P promotes an increase in the proliferation and chemotaxis of HUVEC cells accompanied with enhanced in vitro angiogenesis [113,114]. This data confirms the involvement of molecules such as S1P and its receptors as modulators of angiogenesis and reflects its importance in tumor vascularization.

Considering the importance of ceramides in the regulation of multiple tumor processes, recently the ceramide nanoliposomes effect on mammary tumor cells cultures has been described. In MDA-MB-231 cells, the treatment of C6 ceramides encapsulated in liposomes (ceramide nanoliposomes) was able to significantly increase apoptosis under non-adherent conditions (anoikis), accompanied by an increase in caspase 3/7 activity [115]. In addition, the ceramide nanoliposomes promoted a decrease in cell migration, associated with a diminution in the secretion of IL-6 and IL-8 in MDA-MB-231 cells [115].

Therefore, the interrelation between growth factors and the metabolism of sphingolipids in breast cancer cells has been demonstrated. For example, it has been shown that the treatment of MCF7 mammary tumor cells with epidermal growth factor (EGF) promotes an increase in the expression of sphingosine kinase 1, an enzyme required for cell proliferation and migration [116,117]. Interestingly, in the same cell line, the oestrogen-induced transactivation of EGFR depends on the sphingosine kinase pathway and S1P, a process accompanied by the activation of Src and matrix secretion of metalloproteinases, proteins highly involved in the regulation of migration, invasion, and tumor metastasis [117].

Finally, it is important to highlight the differences between events regulated by ceramides, sphingolipid-metabolites, and S1P. For instance, the presence of high levels of ceramides and their producing enzymes (CerS2) leads to a decrease in the secretion and activity of metalloproteinases, resulting in poorly invasive phenotypes of breast cancer cell lines [118]. In the other hand, the role of S1P as a modulator of cell invasion and metastasis has been demonstrated. In human glioblastoma cells, S1P induces an increase in the secretion and activity of metalloproteinases, leading to the degradation of extracellular matrix, invasion of local tissue and finally, metastasis [119]. Additionally, S1P receptors play an important role in breast tumor development. It has been determined that sphingosine-1-phosphate receptor 1 (S1P_1_) is over-expressed in multiple breast cancer cell lines, positively regulating processes such as tumor migration and invasion. In line with this notion, the down-regulation of S1P_1_ inhibits cell proliferation, colony formation, migration and invasion [120].

Studies show that sphingosine-1-phosphate receptor 3 (S1P_3_) is over-expressed in invasive breast cancer cell lines, resulting in an increase in the expression of COX-2 and in microsomal prostaglandin (PG) E2 synthase, accompanied by a high synthesis of PGE2 and favoring tumor metastasis [121]. S1P_3_ has been involved as a regulator of blood-brain barrier permeability. In the breast cancer brain metastasis, the surrounding astrocytes express high levels of S1P_3_, which regulates and promotes the secretion of CCL2 and IL-6, leading to the relaxation of endothelial cell adhesion and increases the permeability of the blood-brain barrier [122].

This data strongly suggests that the subtle balance between the metabolites and enzymes producing ceramides/sphingolipids in tumor cells leads to the modulation of mammary tumor progression, highlighting the role of these molecules and their receptors as potential targets for breast cancer intervention, specifically associated to metastasis.

## 8. Exosome-Ceramides and the Role in Tissue Communication

Extracellular vesicles are spherical structures surrounded by a bilayer lipid membrane that are found in several body fluids and are secreted by a wide variety of cell types, including tumor cells [123]. According to the International Society for extracellular vesicles, these structures are classified depending on their size and biogenesis in apoptotic bodies, microvesicles, and exosomes [124,125]. Particularly, the exosomes are originated from MVEs, presented average size of 30–100 nm. In different types of cancer, altered levels of exosome release are observed, and their number, composition, and cellular origin depend on the state of the disease [124]. Moreover, it has been shown that exosomes regulate processes of tumor progression, such as proliferation, migration, invasion, metastasis, immunosurveillance, and evasion [126], as well as resistance to chemotherapy, denoting them as an important feature in the study of breast cancer progression [123]. Once secreted, exosomes mediate intercellular communication in autocrine, paracrine, and endocrine pathways. The exosomes modulate the functions of target cells by interacting with surface proteins of vesicles with membrane receptors and being endocyted by the target cell. In this way, the target cells uptake the set of charged molecules present in the exosomes, resulting in modulation of the cellular responses [123,124]. For instance, exosomes secreted by glioma cells transport and transfer EGFRvIII, a constitutively active oncogenic form of EGFR, then acceptor tumor cells show an increase in cell proliferation and survival [127]. Several studies have shown that cancer cells resistant to doxorubicin and cisplatin release high amounts of these molecules through exosomes, therefore their release contributes to tumor cell survival [128,129]. Moreover, cancer cell-derived exosomes contain MMP-2 and MMP-9, enzymes that degrade components of the extracellular matrix, facilitating the invasion of tumor cells into the surrounding tissue (Figure 4) [130]. Furthermore a recent study shows that tumor-secreted exosomes could facilitate metastasis even for tumour cells that lack the capacity to metastasize and this could be regulated by their pattern of integrins expression [131].

Exosome-composition depends on the cells from which they originate, including proteins, nucleic acids (RNA, DNA), and lipids [123,132]. Interestingly, sphingolipids have been associated with the biogenesis and secretion of exosomes. Accumulation of sphingolipids and the high activity of sphingomyelin phosphodiesterase (SMase) generates an increase in levels of free ceramides, associated with the formation of exosomes [124,125,133]. Compared to their parental cells, exosomes secreted by various cell types are enriched with various bioactive lipids such as eicosanoids, fatty acids, ceramides, and sphingomyelin [134,135], therefore, these molecules can be transferred to the acceptor cells. For instance, it has been determined that sphingosine-1-phosphate receptor 2 (S1P_2_) is secreted and transported in exosomes derived from MDA-MB-231 breast cancer cells. Indeed, the exosomes that transport S1P_2_ are taken-up by cancer-associated fibroblasts (CAFs), resulting in an increase in CAFs proliferation dependent on ERK ½ signaling [136].

In addition, S1P is associated with several cellular processes such as angiogenesis, cell growth, motility, and in an important manner can be contained and transported by lipoproteins, the high density lipoproteins (HDL) being the main carrier. Approximately 60% of circulating S1P is associated to HDL, likewise HDL can carry on other sphingolipids such as sphingomyelins and ceramides (C:24) [137,138,139]. HDL is known for its anti-atherogenic function due to its composition of apolipoproteins, among which ApoM is found, whose function is to bind to S1P [140]; both ApoM and S1P are synthesized and secreted by the liver [141]. Although S1P has been considered as a cardioprotective molecule, S1P is described as a possible mediator of HDL-dependent angiogenesis, due to its interaction with receptor S1P_3_, whose activation promotes the expression of VEGFR2 [142]. Therefore, S1P is a moonlighting molecule, although it has a cardioprotective role through angiogenesis activation, this same effect can be detrimental favoring tumor proliferation. In this sense, S1P3 has been found up-regulated in breast cancer cells, a condition associated to increased migration and invasion of metastatic cells induced by an inflammatory environment in a COX-2-PGE_2_-dependent pathway [121].

On the other hand, S1P is capable of activating the matriptase, a serine protease with an important role in tissue remodeling [143], whose overexpression found in breast cancer tissue is associated with a poor prognosis [144] through activation of PAR-2-NF-κB and PI3K-Akt-mTOR, pro-inflammatory and pro-oncogenic signaling, respectively [145]. Likewise, matriptase has been implicated in invasiveness and metastasis in prostate cancer [146,147]. Recently, M69 antibody has been employed in TNBC cells with overexpressed matriptase [148] indicating that this S1P-activated enzyme has an important role on breast cancer therapy.

## 9. Role of Ceramides in Chemoresistance

Ceramides have been proposed as key regulatory molecules in breast cancer chemoresistance, being even considered as sensitizers to chemotherapy. The poor response in chemotherapy is a phenomenon commonly observed in breast cancer, however, is exacerbated in TNBC cells, a breast cancer subtype with a poor prognosis [149,150]. Several cell pathways have been proposed as triggers of neoplastic processes in TNBC, such as CD73, CD133, IMP3, ABCG2, HSF1 and PI3K/AKT/mTOR [150,151,152,153,154,155]. In breast cancer patients studied by Che J., et al., it has been found that after exposition to doxorubicin, cyclophosphamide, fluorouracil or its combination, ceramides levels decreased, indicating that its down-regulation is a common intrinsic response against breast cancer chemotherapy [20]. This subside response of ceramides concentration was determined in mammary tumor tissue, while in untreated women the levels remain unchanged [5]. Specific enzymes of ceramide metabolism have been characterized, UDP-glucose ceramide glucosyltransferase (UGCG) levels were higher after exposition to chemotherapeutic agents as doxorubicin, which lead to decreased ceramide levels [148]. Thus, specific inhibition of UGCG led to higher ceramide levels and this enhanced the sensitivity to doxorubicin and cyclophosphamide in MDA-MB-231 cells [20]. Zhang X. et al., reported that doxorubicin treatment induced the expression of UGCG in MCF-7 cells, an estrogen receptor (ERα) positive model, while this phenomenon is absent in MDA-MB-231 (ERα negative), suggesting an association between the ERα and the UGCG [156].

Moreover, UGCG is involved in multidrug resistance processes, especially in breast and colon cancer [157,158]. For instance, in one study the effects of UGCG on multidrug resistance protein 1 (MDR1) levels was characterized, which concluded that UGCG, specifically Gb3 and Gb4 up-regulates expression of MDR1 through β-catenin signaling, granting doxorubicin and paclitaxel resistance to murine breast cancer cells [159]. In addition, hyper-methylation of CpG islands in 5’ regions of UGCG promoter leads to its inhibition and subsequently to a major sensitivity to chemotherapy in breast cancer cells. In this investigation, authors demonstrated that methylation of UGCG promoter is performed by DNA methyltransferases (DNMTs). The UGCG promoter can be demethylated after the effect of DNMTs inhibitor 5-Aza-dc (Azacitidine), which has been used for therapy at hematological neoplastic processes, indicating the direct relationship between UGCG methylation and cell chemo-sensitivity [160].

Tamoxifen treatments have been reported to increase levels of C16, dHC16, C18, C20, dHC20, dHC22 ceramides in 4T1, MCF-7 and MDA-MB-231 cells, processes accompanied to JNK phosphorylation, caspase-3 and PARP cleavage, both conditions known as markers for cell death, suggesting that tamoxifen also induces cell death in a ceramide-dependent manner [161]. Moreover, tamoxifen shows an antagonist effect over P-gp, a protein with a flippase activity which transports glucosylceramides into Golgi lumen, and this inhibition results in a decreased ceramide glycosylation that leads to increased ceramide levels. Likewise, inhibition of the activity of acid ceramidase by tamoxifen has been reported. Therefore, tamoxifen effects on activation of cell death markers, diminution of ceramide glycosylation, and acid ceramidase inhibition explain its role at enhancing sensitivity to chemotherapy in breast cancer cells [162]. In addition, RIP2, an important mediator of innate immunity, has been recently studied as a modulator of breast cancer survival by Jafaar R., et al., whose results indicate that this kinase confers resistance to ceramide-induced apoptosis in triple-negative breast cancer cells treated with paclitaxel [163]. Thus, RIP2 inhibition can be proposed as a novel target to improve ceramide-induced apoptosis and then, a better antineoplastic drugs response.

## 10. Final Considerations

Ceramides are a species of sphingolipids whose functions have become relevant due to their various intracellular and signaling functions, some of them involved in pathological processes. Sphingolipid metabolism presents moonlighting effects on neoplastic processes due to its multiple metabolites, for instance ceramides being the proapoptotic intermediates, glucosylceramides the inducers of chemoresistance, and S1P the mitogenic sphingolipid [162]. Thus, it is critical to deepen the knowledge about why the increase in ceramides during initial stages of oncogenic processes favors the survival of the tumoral cells, but later becomes cytotoxic, opposed to the early phases. Evidence suggests that ceramide metabolism is associated with the efficacy of breast cancer therapeutics, several reports have evaluated the possibility to use ceramides as a vehicle in cancer treatments. Therefore, a better understanding of the mechanisms by which sphingolipids regulate cancer cell signaling and metabolism will improve the development of therapeutics.

The aim of this work was to describe an integral relationship between ceramide metabolism and breast cancer development and progression, highlighting its importance in the modulation of key processes such as apoptosis, proliferation, EMT, migration, invasion, and metastasis, resulting in a highly comprehensive and novel work that contributes to the better understanding of tumor ceramides metabolism. Therefore, this work represents a new approach to mechanisms that trigger cancer development and the role of ceramides. 

Ceramides function on cell apoptosis has been demonstrated, then these lipids are closely related to mitochondrial structure alterations, facilitating the flow of pro-apoptotic proteins from the intermembrane space into the cytoplasm; also, they exert intracellular effects on insulin signaling induced by meta-inflammation. Therefore, ceramides can be visualized as a hub in the cellular metabolism by functioning as molecules with dual roles, as under stress conditions, which lead to inflammation, they regulate several functions such as apoptosis, and even subtle phenomena in the regulation of cellular cancer signaling mechanisms. The specific conditions in which these lipids will become pathological molecules is a subject pending elucidation.

## Abbreviations

ER	Endoplasmic reticulum
FFA	Free fatty Acids
SFA	Saturated Fatty Acids
ECT	Electron transport chain
ROS	Reactive oxygen specie
DAGs	Diacylglycerols
INSR	Insulin receptor
SPT	Serin-palmitoyl acyltransferase
CerS	Ceramide synthase
NLRP3	NOD-like receptor family pyrin domain containing 3
MVEs	Multivesicular endosome
ESCRT-I	Endosomal sorting complex required for transport
OMM	Outer mitochondrial membrane
DES	Dihydroceramide desaturase
CERT	Ceramide transfer protein
SMase	Sphingomyelinase
UPR	Unfolded protein response
PACS-2	Phosphofurin acidic cluster sorting protein 2
IMM	Inner mitochondrial membrane
ERO-1α	Endoplasmic reticulum oxidoreductase-1 alpha
PD-L1	Programmed cell death-ligand 1
PAC-1	Compound 1 activator of procaspase
Mcl-1	Myeloid cell leukemia-1 Mcl-1
VDAC-1	Voltage-dependent anion-selective channel 1
H2K	Hexokinase-2
GPCR	G Protein-coupled receptor
S1P	Sphingosine-1-phosphate
SphK	Sphingosine kinase
EMT	Epithelial-mesenchymal transition
CK	Cytokeratin
Gg4	Galpβ1-3GalpNAcβ1-4Galpβ1-4Glcpβ
TNBC	Triple negative breast cancer
EGF	Epidermal growth factor
Erα	Estrogen receptor
UGCG	UDP-glucose ceramide glucosyltransferase
5-Aza-dc	Azacitidine
MAMs	Mitochondria-associated ER membranes
MDR1	Multidrug resistance protein 1
DNMTs	DNA methyltransferases
